# Abnormal Myocardial Contractility After Pediatric Heart Transplantation by Cardiac MRI

**DOI:** 10.1007/s00246-017-1642-5

**Published:** 2017-05-30

**Authors:** Heynric B. Grotenhuis, Emile C. A. Nyns, Paul F. Kantor, Anne I. Dipchand, Steven C. Greenway, Shi-Joon Yoo, George Tomlinson, Rajiv R. Chaturvedi, Lars Grosse-Wortmann

**Affiliations:** 1 Department of Paediatrics, Labatt Family Heart Centre, The Hospital for Sick Children, University of Toronto, 555 University Avenue, Toronto, ON M5G 1X8 Canada; 2 Department of Pediatric Cardiology, Stollery Children’s Hospital, Edmonton, AB Canada; 3 Departments of Paediatrics and Cardiac Sciences, Alberta Children’s Hospital, University of Calgary, Calgary, AB Canada; 4 Department of Diagnostic Imaging, The Hospital for Sick Children, University of Toronto, Toronto, ON Canada; 5 Department of Medicine, Toronto General Hospital and Mt. Sinai Hospital, University of Toronto, Toronto, ON Canada

**Keywords:** Acute cellular rejection, Pediatric, Heart transplant, Myocardial deformation, Magnetic resonance imaging

## Abstract

Acute cellular rejection (ACR) compromises graft function after heart transplantation (HTX). The purpose of this study was to describe systolic myocardial deformation in pediatric HTX and to determine whether it is impaired during ACR. Eighteen combined cardiac magnetic resonance imaging (CMR)/endomyocardial biopsy (EMBx) examinations were performed in 14 HTX patients (11 male, age 13.9 ± 4.7 years; 1.2 ± 1.3 years after HTX). Biventricular function and left ventricular (LV) circumferential strain, rotation, and torsion by myocardial tagging CMR were compared to 11 controls as well as between patients with and without clinically significant ACR. HTX patients showed mildly reduced biventricular systolic function when compared to controls [LV ejection fraction (EF): 55 ± 8% vs. 61 ± 3, *p* = 0.02; right ventricular (RV) EF: 48 ± 7% vs. 53 ± 6, *p* = 0.04]. Indexed LV mass was mildly increased in HTX patients (67 ± 14 g/m^2^ vs. 55 ± 13, *p* = 0.03). LV myocardial deformation indices were all significantly reduced, expressed by global circumferential strain (−13.5 ± 2.3% vs. −19.1 ± 1.1%, *p* < 0.01), basal strain (−13.7 ± 3.0% vs. −17.5 ± 2.4%, *p* < 0.01), mid-ventricular strain (−13.4 ± 2.7% vs. −19.3 ± 2.2%, *p* < 0.01), apical strain (−13.5 ± 2.8% vs. −19.9 ± 2.0%, *p* < 0.01), basal rotation (−2.0 ± 2.1° vs. −5.0 ± 2.0°, *p* < 0.01), and torsion (6.1 ± 1.7° vs. 7.8 ± 1.1°, *p* < 0.01). EMBx demonstrated ACR grade 0 R in 3 HTX cases, ACR grade 1 R in 11 HTX cases and ACR grade 2 R in 4 HTX cases. When comparing clinically non-significant ACR (grades 0–1 R vs. ACR 2 R), basal rotation, and apical rotation were worse in ACR 2 R patients (−1.4 ± 1.8° vs. −4.2 ± 1.4°, *p* = 0.01 and 10.2 ± 2.9° vs. 2.8 ± 1.9°, *p* < 0.01, respectively). Pediatric HTX recipients demonstrate reduced biventricular systolic function and decreased myocardial contractility. Myocardial deformation indices by CMR may serve as non-invasive markers of graft function and, perhaps, rejection in pediatric HTX patients.

## Introduction

Advances in donor and recipient selection, surgical techniques, and medical management have substantially improved survival of children after heart transplantation (HTX) [1–3]. Despite these encouraging developments, cardiovascular health remains compromised in many children and adolescents after HTX. Acute cellular rejection (ACR)—although its prevalence has decreased over the years—remains an important cause of morbidity and graft loss and is one of the factors compromising the long-term functional integrity of the graft [1–5]. Acute cellular rejection is a risk factor for graft loss, cardiac dysfunction, coronary vascular disease, and mortality. Therefore, surveillance for ACR is crucial, especially since many patients with ACR are asymptomatic [2, 6]. Presently, endomyocardial biopsy (EMBx) is the gold standard to detect subclinical ACR in HTX patients, as serologic and echocardiographic markers are not sufficiently sensitive and/or specific [1–6]. However, the radiation burden, the invasive nature of the test, as well as the need for sedation or general anaesthesia are barriers to EMBx, especially in children [1–6]. Suitable alternative tests for ACR are therefore desirable for pediatric HTX patients [2].

Abnormal left ventricular (LV) torsion and biventricular systolic dysfunction have been reported in pediatric HTX recipients [5, 7–11], and reduced LV ejection fraction (EF) has been proposed as a non-invasive marker of ACR in children [7]. However, little is known about LV myocardial deformation after pediatric HTX in relationship with graft rejection. Cardiac magnetic resonance imaging (CMR) is the reference method for biventricular volumetry and EF, and is also suited to assess LV strain, rotation, and torsion [8, 12–19]. The purpose of this study was to assess, in detail, systolic myocardial function in pediatric HTX recipients by CMR.

## Materials and Methods

### Patients

This is a single-center, prospective cross-sectional cohort study. The study was approved by the institutional research ethics board and informed consent was obtained from all participants and/or their legal guardians at the time of enrolment. All children and adolescents after HTX who underwent a clinically indicated EMBx between April 2010 and March 2011 were invited to undergo a CMR scan on the same day. EMBx were requested either as part of routine ACR surveillance (17 EMBx) or as follow-up after treatment of ACR (1 EMBx). None of the EMBx were performed because of a suspicion of ACR. CMR examination was conducted immediately before the EMBx in all participants, either in an awake cooperative patient or under the same anaesthesia as the EMBx. Exclusion criteria were general contraindications to undergo CMR, arrhythmia interfering with CMR acquisition as well as poor CMR image quality. Family members of patients with arrhythmogenic right ventricular cardiomyopathy (ARVC) who underwent CMR screening for the condition were recruited as controls. Individuals with any signs or symptoms of ARVC, including abnormal CMR findings, were excluded from the control cohort. Patient charts were reviewed for age of the organ donor, ischemia time of the donor heart, bypass time at HTX, and immunosuppressive medications at time of CMR/EMBx. Where available, the reports of fluoroscopic angiography (within 1 year of CMR/EMBx) were reviewed for signs of cardiac allograft vasculopathy (CAV) in accordance with the International Society for Heart and Lung Transplantation (ISHLT) grading system [20]. At the authors’ institution, routine two-yearly screening by coronary angiography for cardiac allograft vasculopathy (CAV) begins between 2 and 5 years post-HTX, depending on age, size, and risk factors for development of CAV.

### Cardiovascular Magnetic Resonance Imaging

CMR examinations were performed on a 1.5-T scanner (‘Avanto’ Siemens Medical Solutions, Erlangen, Germany). For ventricular volumetry, a short-axis cine stack was acquired with a steady-state free precession technique, using the following parameters: temporal resolution to allow 20 phases per cardiac cycle, repetition time 2.97 ms, echo time 1.34 ms, field of view 260 mm, flip angle 78°, voxel size 1.3 × 1.3 × 5 mm, and variable spacing to cover both ventricles with 12–13 slices.

Myocardial tagging of the LV was obtained at three levels in short axis (base, mid-ventricular, and apical). Imaging parameters included a temporal resolution sufficient to allow 14 or more true phases per cardiac cycle, repetition time 11.7 ms, echo time 6.6 ms, flip angle 7°, voxel size 2.4 × 1.6 × 5 mm, and grid size 6 mm.

Biventricular end-diastolic volume (EDV), end-systolic volume (ESV), and EF, as well as LV mass were analyzed in the usual fashion [21], using commercially available software (‘QMass’ version 7.2; Medis, Leiden, The Netherlands). Indexation to body surface area (BSA) was performed using the Mosteller formula (BSA = [*H* × *W*/3600]^½^), where *H* is the height in centimeters, and *W* is the weight in kilograms. LV mass was also indexed for LV EDV, to allow for comparison of LV mass relative to LV size.

**Fig. 1:**
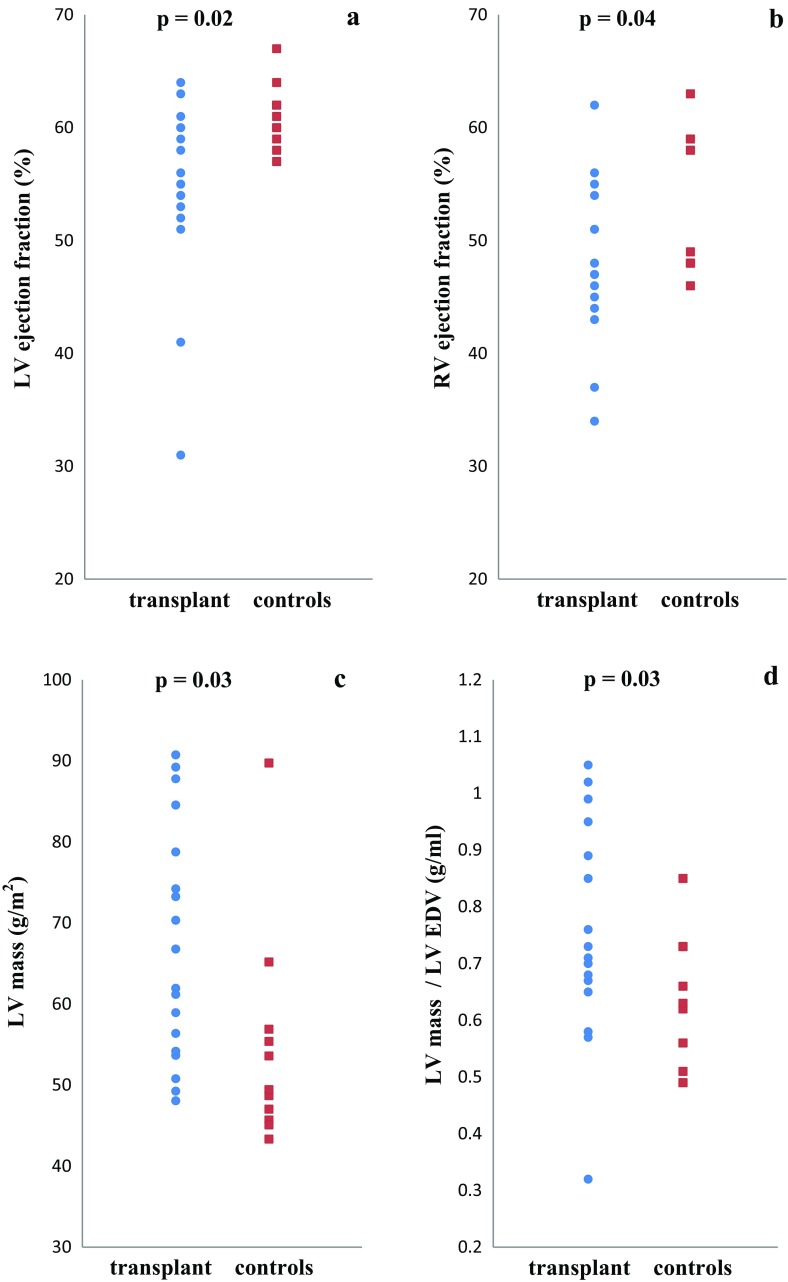
**a**–**d** Left ventricular (LV) ejection fraction, right ventricular ejection fraction, LV mass, and LV mass-to-volume ratio in controls and transplant recipients. LV ejection (**a**) and right ventricular (RV) ejection fraction (**b**) are significantly lower in heart transplant patients, but show strong overlap between healthy controls and heart transplant patients. Indexed LV mass (**c**) and LV mass-to-end-diastolic volume (EDV) ratio (**d**) were also significantly increased in transplant patients

The analysis of CMR tagging images was performed by a single observer with 10 years of experience in CMR, who was blinded towards the patients’ clinical status, and the results of the endomyocardial biopsies. The images were analyzed using ‘HARP’ software (Diagnosoft, Palo Alto, California, USA) [14, 16]. Transmural circumferential Lagrangian strain and rotation were measured at the basal, mid-ventricular, and apical levels of the LV, as was described elsewhere [14, 16]. In brief, strain is defined as the relative deformation of myocardium, with negative values denoting shortening [8, 12]. Rotation is defined as the angular change of the LV in a short-axis plane about the LV long axis [12]. For this, epicardial and endocardial contours at all 3 levels were traced in mid-systole [14, 16]. An automated contour detection algorithm detected the contours in the remaining phases of the cardiac cycle. The myocardium on each short-axis circumference was divided into 24 segments (Fig. 1) [14, 16]. Non-tracking segments were excluded from analysis [14]. By convention, the assessment of LV strain and rotation was considered feasible when the myocardial borders were auto-tracked correctly in 13 or more of the 24 segments. Peak strain and rotation were obtained as the differences between the first and the last systolic image, with end-systole defined as the phase with the smallest LV cross-sectional area at the mid-ventricular level [14, 16]. Circumferential strain was calculated per slice as the average of all included segments [14, 16]. Global circumferential strain was calculated as the average strain at the basal, mid-ventricular, and apical levels [14, 16]. All results on circumferential strain will be referred to a ‘strain’ from here on. Rotation was calculated per slice in a similar way to that described for the strain assessment, but only for the LV apex and base [14, 16–19]. Clockwise rotation as viewed from the apex was expressed as a negative angle [17–19]. LV twist was calculated as the net difference between LV apical and basal rotation [12, 17–19]. As the intersubject comparability of twist is limited in the pediatric population due to considerable interindividual variations in heart length and diameter, torsion is preferred to twist as it corrects for cardiac size [19]. Torsion was calculated as follows [17–19]: (apical rotation − basal rotation) × (apical radius + basal radius)/(2 × distance between apex and base). The distance between the apical and basal slices was derived from short-axis stack as the distance between the two slice locations. The radii of base and apex were calculated as the average of 3 cross-sectional diameters of the tagging contours in the end-systolic phase, divided by 2.

### Endomyocardial Biopsy

Cardiac catheterization for EMBx was performed using a right internal jugular venous approach. Four to five endocardial samples were taken from the right ventricular (RV) aspect of the interventricular septum. Experienced cardiac pathologists, who were blinded towards the imaging findings and the patients’ clinical status, performed the histological analysis of the specimens. Biopsies were clinically graded according to the 2005 nomenclature of the ISHLT for ACR as 0, 1 R, 2 R, or 3 R [22] and antibody-mediated rejection (AMR) as AMR 0, AMR 1 [23].

### Statistical Analysis

Analyses were done in both a SPSS software package (version 20, Chicago, Illinois, USA) and R 3.1.2 [24]. All data are presented as mean ± standard deviation, unless stated otherwise. Independent samples *T* test was used to express differences between patients and control subjects. Correlation between variables was assessed using Pearson’s correlation coefficient. Statistical significance was indicated by *p* < 0.05.

## Results

Fourteen pediatric HTX recipients (11 male, 3 female) and 11 controls (4 male, 7 female) participated in the study. Donor age was 15.1 ± 9.3 years (8 days to 29 years). Ischemic time and bypass time at HTX had been 209 ± 73 min and 121 ± 59 min, respectively. Immune suppression consisted of Tacrolimus and mycophenolate mofetil in 13 HTX patients, supplemented by Sirolimus in one and prednisone in another HTX patient. In addition, 11 HTX patients received statins and 10 HTX patients received antihypertensive medication. Two patients were studied on more than one occasion: one patient underwent two CMR/EMBx investigations (5 months apart) and another patient had 4 combined CMR/EMBx investigations (intervals of 1, 4, and 7 months, respectively). Coronary angiography was performed in 9 of 14 patients, showing no evidence of CAV (all showed CAV 0).

A total of 18 combined CMR/EMBx investigations were analyzed and results are summarized in Table 1. General anaesthesia was performed in three combined CMR/EMBx procedures. In the other 15 CMR/EMBx procedures, patients were awake during the CMR and received sedation for the EMBx. Six patients underwent early EMBx after HTX (within 6 months) with an interval between HTX and EMBx between 12 days and 24 weeks. Acute cellular rejection grade 0 R was observed in three cases, ACR grade 1 R in 11 cases and ACR grade 2 R in 4 cases. No case of ACR grade 3 R was detected. The HTX patient with 2 CMR/EMBx investigations showed ACR grade 1 R on both occasions. The HTX patient with 4 repeated investigations demonstrated ACR grade 1 R on the first 2 EMBx and ACR grade 2 R on the last 2 EMBx. Antibody-mediated rejection was not observed in this study.

**Table 1:** Demographics and magnetic resonance results in transplant recipients and controls

	HTX patients (*n* = 18)	Controls (*n* = 11)	*p* value
Age at CMR (years)	13.9 (4.7) (2.4–17.9 years)	13.1 (3.2) (7.6–18.0 years)	0.64
Interval HTX to CMR (years)	1.2 (1.3) (12 days–5.0 years)		
Height at CMR (cm)	155 (27)	156 (15)	0.94
Weight at CMR (kg)	64.0 (30.6)	49.1 (11.0)	0.13
Body surface area at CMR (m^2^)	1.61 (0.52)	1.45 (0.22)	0.33
Rejection grade 1 R	11		
Rejection grade 2 R	4		
LV EF (%)	55 (8)	61 (3)	0.02
LV EDV (ml/m^2^)	90 (23)	88 (14)	0.82
LV ESV (ml/m^2^)	42 (20)	35 (7)	0.25
LV mass (g/m^2^)	67 (14)	55 (13)	0.03
LV mass/LV EDV (g/ml)	0.78 (0.21)	0.62 (0.12)	0.03
RVEF (%)	48 (7)	53 (6)	0.04
RV EDV (ml/m^2^)	104 (22)	97 (12)	0.30
RV ESV (ml/m^2^)	54 (15)	45 (9)	0.10
Global strain (%)	−13.5 (2.3)	−19.1 (1.1)	<0.01
Strain base (%)	−13.7 (3.0)	−17.5 (2.4)	<0.01
Strain mid-ventricular (%)	−13.4 (2.7)	−19.3 (2.2)	<0.01
Strain apex (%)	−13.5 (2.8)	−19.9 (2.0)	<0.01
Rotation base (°)	−2.0 (2.1)	−5.0 (2.0)	<0.01
Rotation apex (°)	8.6 (4.1)	8.3 (3.0)	0.84
LV torsion (°)	6.1 (1.7)	7.8 (1.1)	<0.01

Results are expressed as mean values and standard deviations between brackets

*CMR* cardiac magnetic resonance, *EDV* end-diastolic volume, *EF* ejection fraction, *ESV* end-systolic volume, *HTX* heart transplant, *LV* left ventricle, *RV* right ventricle

**Fig. 2:**
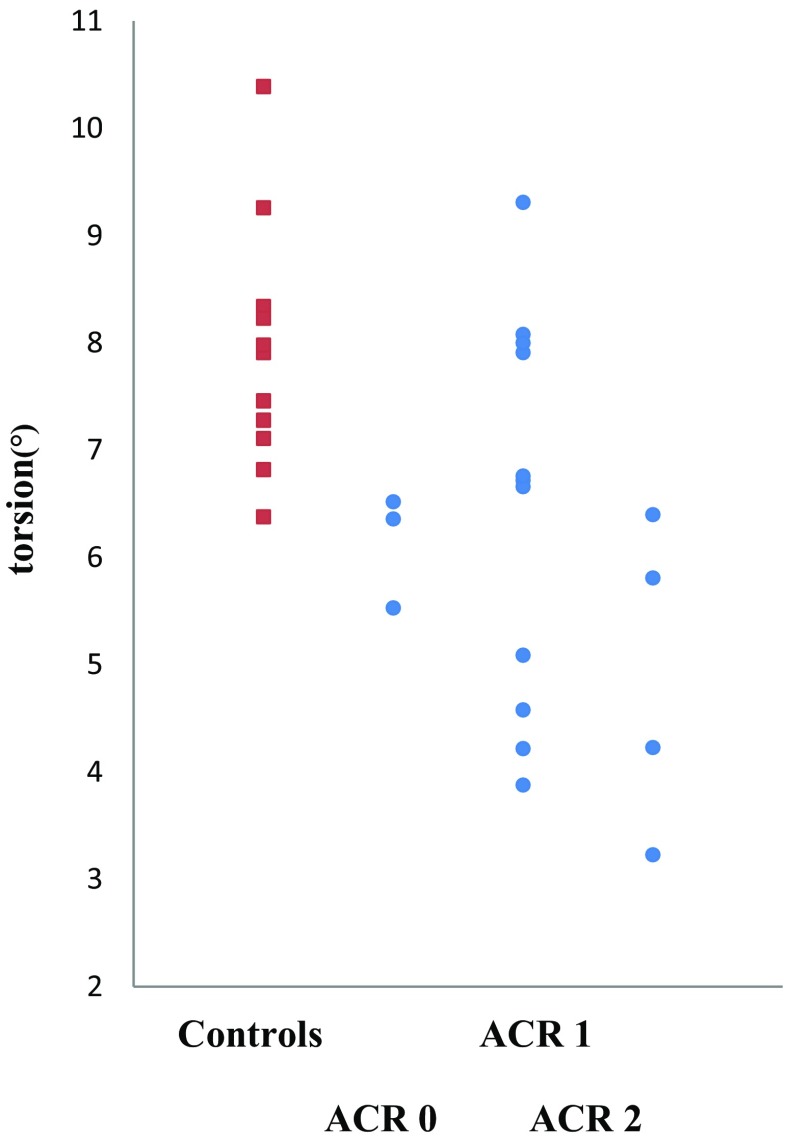
Torsion in controls and transplant recipients. Acute cellular rejection (ACR) versus torsion in transplant patients (*filled circle*) and controls (*filled square*)

The 18 CMR studies demonstrated mildly reduced left and right ventricular EFs in HTX patients when compared to controls (*p* = 0.02 and *p* = 0.04, respectively) (Table 1; Fig. 1). Patients after HTX also showed decreased global circumferential strain, strain at all three LV short-axis levels, basal rotation, and LV torsion when compared with controls (*p* < 0.01 for all) (Table 1; Fig. 2). Seven HTX patients had LV EF <55% (range 31–54%) and four HTX patients had RV EF <45% (range 34–44%). No differences in biventricular dimensions (indexed EDV and ESV) were observed between patients and controls. HTX patients showed increased indexed LV mass when compared to controls (*p* = 0.03) (Fig. 1c), also when LV mass was corrected for LV EDV (*p* = 0.03) (Table 1; Fig. 1d).

**Fig. 3:**
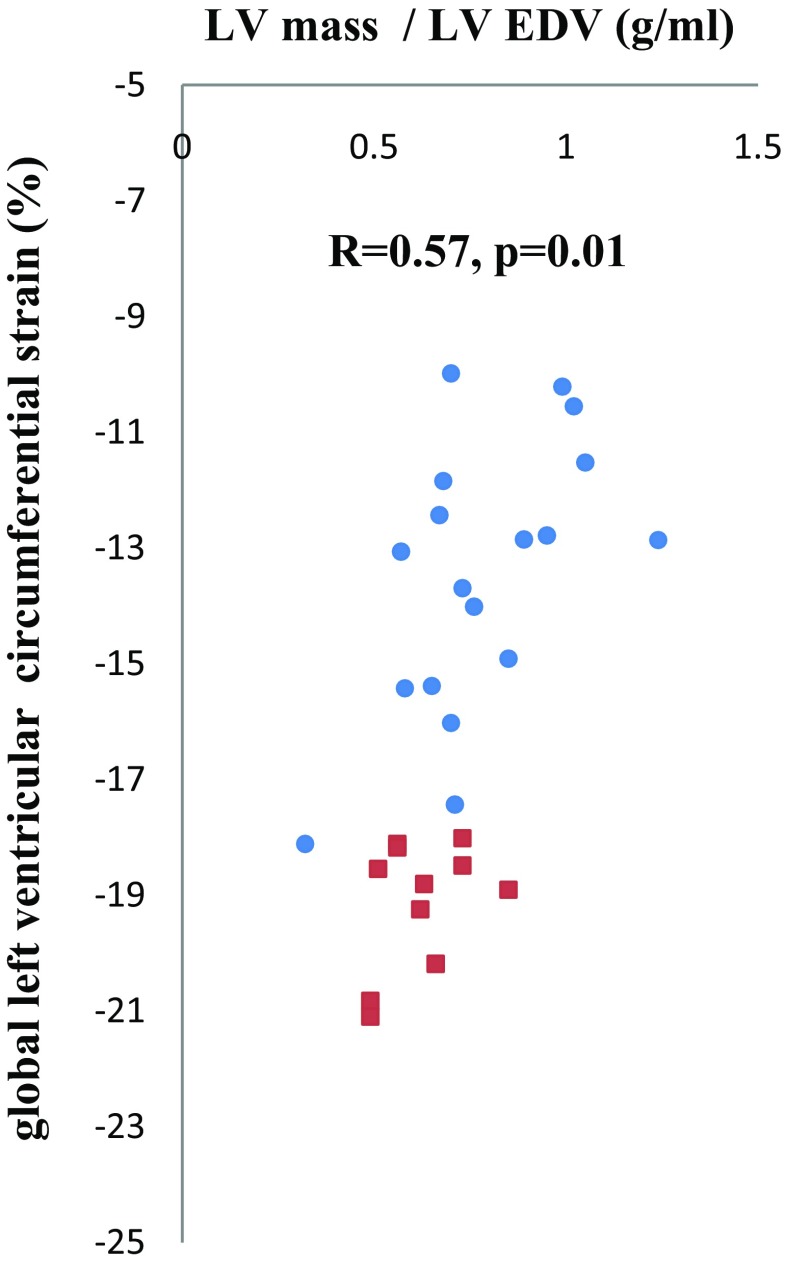
Left ventricular mass-to-end-diastolic volume ratio versus global strain in transplant patients and controls. LV mass-to-end-diastolic volume (EDV) ratio versus global strain in transplant patients (*filled circle*) and controls (*filled square*)

Increased LV mass correlated with apical strain (*R* = 0.53, *p* = 0.03), while LV mass/EDV correlated with global strain (*R* = 0.57, *p* = 0.01) (Fig. 3), mid-ventricular strain (*R* = 0.55, *p* = 0.02), and apical strain (*R* = 0.61, *p* < 0.01). Reduced LV torsion correlated with basal strain (*R* = 0.58, *p* = 0.01) and apical rotation (*R* = 0.66, *p* < 0.01). Myocardial deformation and biventricular EFs were not associated with ischemic time and bypass time at HTX in our study.

When HTX patients were stratified by early (<6 months, 6 CMR/EMBx studies) versus late (≥6 months, 12 studies) CMR/EMBx after HTX, strain at mid-ventricular and apical levels were better in the ‘late’ group (−11.7 ± 2.1 vs. −14.5 ± 2.5, *p* = 0.02 and −11.8 ± 2.6 vs. −14.5 ± 2.5, *p* = 0.02, respectively). Late and early time points after HTX did not differ with regards to rotation, torsion, ventricular volumes, LV mass, and ejection fractions. When comparing only patients without significant rejection (ACR 0 or 1 R), mid-ventricular and apical strain were no longer statistically different between early and late after transplant, although a trend prevailed (*p* = 0.06 for both).

Patients with clinically non-significant ACR (grades 0–1 R) were compared with HTX patients with more significant ACR (grade 2 R), showing that basal rotation and apical rotation were worse in patients with ACR 2R (−1.4 ± 1.8° vs. −4.2 ± 1.4, p = 0.01 and 10.2 ± 2.9° vs. 2.8 ± 1.9, *p* < 0.01, respectively), but not for biventricular dimensions and ejection fractions, and the other myocardial deformation indices. When comparing only the 14 first CMR studies between patients with non-significant ACR and those with significant ACR, basal and apical rotation remained worse in patients with ACR 2 R (*p* < 0.01 and *p* < 0.01, respectively).

## Discussion

Abnormal myocardial deformation and systolic dysfunction have been reported in pediatric HTX recipients. However, the extent of systolic dysfunction and myocardial deformation abnormalities after pediatric HTX in relationship with graft rejection is unknown. The current CMR study compares ventricular systolic function and myocardial deformation between HTX patients and controls, and describes their relationship with the presence of rejection as determined by endomyocardial biopsy in pediatric HTX recipients. The study adds the following to our understanding of cardiovascular health in pediatric patients after HTX:

- Children after HTX have decreased biventricular systolic function;
- They show evidence of decreased myocardial contractility as evidenced by abnormal circumferential strain, rotation, and torsion; and
- LV rotation is reduced with increased severity of ACR.

### Ventricular Ejection Fraction and LV Mass

On average, LV and RV EFs were mildly reduced in HTX recipients compared to controls, although most children’s EFs remained within the normal range. The etiology of the reduced global ventricular systolic function is unclear: transient organ ischemia during transplantation, reperfusion injury, cardiotoxic medications, CAV, and ACR have all been named as possible contributors to impaired ventricular function [1–5]. Studies investigating the suitability of LV EF as a marker of ACR have yielded mixed results in adults and children [2, 5, 6, 25, 26]. In the present study, LV and RV EF did not distinguish between clinically significant and no ACR [2, 5, 6, 26]. Patients had larger LV mass and increased mass-to-volume ratio, the latter suggesting that the increased LV mass is not merely the result of larger graft organs and a donor-recipient mismatch. Another possibility is that the increased LV ‘mass’ by CMR is a reflection of edema, rather than myocyte hypertrophy [1–5, 27]. However, cases with ACR 2R at the time of CMR did not have higher LV mass or mass-to-volume ratio as compared to controls.

### Circumferential Strain, Rotation, and Torsion

Not only global ventricular function is decreased in HTX recipients, myocardial deformation as expressed by abnormal circumferential strain, rotation, and torsion is also impaired: we found reduced global circumferential strain as well as strain at all three LV short-axis levels, corroborating previous reports [1, 3–5, 26]. Myocardial strain has been proposed as a sensitive marker of subclinical changes in myocardial performance in a variety of conditions; abnormalities in strain often precede a decline in EF [27, 28]. Miller and colleagues witnessed an association of reduced circumferential strain by CMR tagging in adult HTX recipients with clinically significant ACR (grade 2 R or higher) [26]. The mid-ventricular circumferential strain values that they report are comparable to those that we found in our pediatric cohort. In their study, a reduction of mid-ventricular strain was associated with CMR markers of myocardial edema including myocardial T1 and T2 relaxation times, suggesting myocardial edema due to inflammation as a possible cause of functional demise [26]. In a rodent transplant model, areas of abnormal circumferential strain by CMR tagging corresponded to regions of macrophage infiltration [29]. In the present study, we did not measure CMR markers of myocardial edema, but did not find differences in strain with ACR 2 R as compared to 0 R and 1 R.

Controversy persists over which strain dimension is primarily decreased after HTX and affected the most during ACR. In contrast to our and Miller’s studies which focused on circumferential strain [26], several echocardiography-based reports—using speckle-tracking and tissue Doppler imaging—described predominantly reduced *longitudinal* LV strain and relatively preserved *circumferential* LV strain after pediatric and adult HTX [8, 9, 28, 30, 31]. Other studies in adults—both by echocardiography and CMR tagging—found *longitudinal* and *circumferential* strain to be equally affected by clinically significant ACR (>grade 2 R) [6, 25, 26, 31]. Apart from ACR, it is possible that other factors impact myocardial mechanics. Some of these factors are related to the transplant surgery, although myocardial deformation and biventricular EF were not associated with ischemic and bypass times at HTX in our study. In clinical practice, patients often experience some degree of cardiac dysfunction early after transplantation; most regain normal systolic function after several weeks to months. In agreement with this clinical observation, patients who underwent CMR within the first 6 months after HTX showed lower mid-ventricular and apical circumferential strain, indicating decreased contractility early after transplantation. Whether this improvement in contractility reflects favorable myocardial remodeling or is primarily reflective of the higher incidence of rejection early after HTX remains to be studied.

Torsion describes the wringing motion of the LV cavity secondary to rotation at the base and at the apex in opposite directions. In comparison with rotation, torsion is a more comprehensive parameter of the LV’s twisting motion as it represents the spiral orientation of LV fibers, incorporating both basal and apical rotation [8, 10, 11]. It has an important role for both systolic and diastolic function: ejection during systole is assisted by rotational deformation, whereas rapid untwisting during early diastole enhances LV suction by augmenting intraventricular pressure gradients allowing for ventricular filling at a relatively low left atrial filling pressure [9]. Donofrio et al. were the first to report reduced torsion in pediatric HTX recipients by use of CMR [8]. Similarly, the current study revealed reduced LV torsion, mostly due to reduced rotation at the base. The etiology of abnormal torsion and why apical rotation appears to be relatively preserved, is ultimately unclear. Incomplete or heterogeneous cardiac re-innervation after cardiac denervation of the transplanted heart, rejection, and allograft vasculopathy have all been proposed as factors in decreased rotation [8–11].

Donofrio et al. did not relate rotation and torsion to the presence of ACR [8]. In the cohort studied here, LV basal and apical rotation were reduced with increasing degree of ACR, corroborating reports in adults which have described links between rotation and ACR, using both echocardiography and CMR [7, 8, 11, 32]. Sato et al. found that reduced torsion by echocardiography of at least 25% as compared to a patient’s baseline predicted biopsy-proven ACR ≥ 2R ISHLT in adult HTX recipients [32]. In their study, LV torsion returned to baseline within an average of nine days after immunosuppression was augmented.

## Limitations

The principal limitation of this study is—as discussed—the small sample size, particularly the group of HTX patients with grade 2 R ACR, which limits the generalizability of the results. Two patients were studied on more than one occasion which may have influenced the comparison between patients between controls and HTX and between those with significant ACR and those with ACR 0/1 R. However, most of the differences between patients and controls persisted when only one CMR per patient was analyzed. In summary, the observations in this study are hypothesis-generating and must be substantiated in larger studies. Furthermore, while EMBx is the gold standard for the diagnosis of rejection, it may not be reflective of the LV myocardium. This principal shortcoming of EMBx for rejection surveillance may have obscured further associations between CMR markers and rejection.

## Conclusions

Pediatric HTX recipients have mildly reduced biventricular systolic function and abnormal myocardial deformation indices, expressed by impaired LV circumferential strain, rotation, and torsion. These indices may serve as markers of graft function in pediatric HTX patients. Left ventricular rotation is reduced with increased severity of ACR. Studies in larger populations are necessary to clarify whether CMR metrics of myocardial contractility can be used to screen for ACR.
